# Adult secondary hemophagocytic lymphohistiocytosis

**DOI:** 10.1002/jha2.113

**Published:** 2020-10-13

**Authors:** Antonina Obayo, Karishma Sharma, Caroline Mithi, Malkit Riyat, Anne Mwirigi

**Affiliations:** ^1^ Department of Medicine Faculty of Health Sciences Aga Khan University Medical College of East Africa Nairobi Kenya; ^2^ Department of Haematology and Oncology, Faculty of Health Sciences Aga Khan University Medical College of East Africa Nairobi Kenya

**Keywords:** hemophagocytic lymphohistiocytosis, HLH‐2004 diagnostic criteria, systemic lupus erythematosus

## Abstract

**Background:**

Hemophagocytic lymphohistiocytosis (HLH) is a syndrome of excessive inflammation and tissue destruction due to abnormal immune activation. HLH carries a very high mortality, and while delays in patients’ presentation to hospital, time to suspicion of HLH, investigation, and initiation of therapy all play a part, mortality remains high even with timely diagnosis and treatment. Classical manifestations of HLH include persistent fever, cytopenias, and liver dysfunction.

**Case presentation:**

We present four cases of secondary HLH, highlighting the demographic and clinical characteristics of these patients, underlying triggers (including systemic lupus erythematosus, lymphoproliferative disorders, and leishmaniasis), together with challenges associated with the diagnosis and treatment of this rare disorder and a brief review of literature.

**Conclusion:**

HLH has protean manifestations and requires a high index of suspicion as it can be a great clinical masquerader. Mortality due to multiorgan failure is often high even with early recognition and treatment.

## 1 INTRODUCTION

Hemophagocytic lymphohistiocytosis (HLH) is a syndrome of exaggerated inflammatory response and tissue destruction due to abnormal immune activation [1]. It can occur as a familial or sporadic disorder, and may be triggered by various immune activating events.

Primary HLH or familial hemophagocytic lymphohistiocytosis (FHL) refers to HLH caused by gene mutations and presents in childhood. Identifiable causes include the following mutations: 9q21.3‐22, PRF‐1, UNC 13D, STX 11, and STX BP2, Chediak‐Higashi syndrome (CHS‐ 1/LYST), Griscelli syndrome type 2 (RAB 27A), Hermansky‐Pudlak syndrome type 2 (AP3B1), and the X‐linked lymphoproliferative syndromes XLP‐1 (SAP) and XPL‐2 (XIAP) [2].

Secondary (sporadic, acquired) HLH occurs in patients without a known familial mutation, in some of whom a clear trigger for development of HLH can be identified. Acquired or secondary cases of HLH can be broadly triggered by malignancy, infectious, or autoimmune etiology [2, 3, 4]. Commonly, these include Epstein‐Barr virus (EBV), cytomegalovirus (CMV), lymphoproliferative disorders and connective tissue disorders, such as systemic lupus erythematosus (SLE).

## 2 METHODS AND MATERIALS

We conducted a retrospective review of the patient charts of four patients, who were diagnosed with HLH at the Aga Khan University Hospital, Nairobi between 2018 and 2019. The diagnosis of HLH was based on the HLH‐2004 guidelines that require five of eight criteria to qualify for a diagnosis of HLH.

These criteria include fever, cytopenias affecting two lineages (hemoglobin < 9 g/dL, platelets < 100^9^/L, neutrophil count < 1^9^/L), hypertriglyceridemia and/or hypofibrinogenemia, hemophagocytosis on biopsy of bone marrow, spleen or lymph node, hyperferritinemia > 500 ng/mL, impaired natural killer (NK) cell activity, and elevated CD 25 (soluble interleukin 2 receptor).

## 3 CASE PRESENTATION AND RESULTS

### 3.1 Case A

A 30‐year‐old male patient was referred to our outpatient department following treatment for neutropenic sepsis and bone marrow failure at a peripheral facility. He had been unwell for 2 months prior, with fatigue, unexplained weight loss, and fevers as the predominant symptoms. There had been severe anemia necessitating blood transfusion, easy bruising, and evidence of mucosal hemorrhage, with gum bleeding and frank haematuria.

Initial assessment revealed temperature of 39°C, heart rate (HR) of 80/minute, respiratory rate (RR) of 20/minute, and blood pressure (BP) of 90/60 mm Hg. The physical examination was significant for hepatosplenomegaly. Initial investigations at time of admission are summarized in Table 1.

**TABLE 1:** Initial investigations at admission for patient A. Abbreviations: ALP, alkaline phosphatase; ALT, alanine aminotransferase; AST, aspartate aminotransferase; GGT, gamma‐glutamyl transferase; LDH, lactate dehydrogenase

Initial investigation	Value	Reference range
Hemoglobin	8.6 g/dL	11.5‐16.5
MCV	79.60 fL	77‐93
MCH	27.40 pg	27‐33
Hematocrit	22.2%	37‐47
Reticulocyte count	0.11%	0.2‐2
Total leucocyte count	0.85 × 10^9^/L	4‐10
Peripheral blood film	Target cells and tear drop cells. Markedly reduced platelets. No schistocytes.	
Platelet count	4.00 × 10^9^/L	150‐400
Lactate dehydrogenase	890 U/L	135‐214
AST	186.60 U/L	0‐35
ALT	355.40 U/L	0‐35
ALP	171.00 U/L	0‐98
GGT	115 U/L	0‐35
Total bilirubin	43 U/L	0‐21
Direct bilirubin	31 U/L	0‐3.4
Albumin	23.5 g/L	35‐55
Ferritin	16 731 ng/mL	13‐150
Serum potassium	3.55 mmol/L	3.3‐5.4
Serum creatinine	50 Umol/L	58‐96
Serum bicarbonate	20.5 mmol/L	22‐29
Serum sodium	141 mmol/L	135‐145
Blood urea nitrogen	9.6 mmol/L	2.1‐7.1
Hepatitis C antibody	Negative	
Hepatitis B surface antigen	Negative	

A computerized tomography (CT) scan of the chest and abdomen revealed features of pulmonary edema, scattered ground glass opacities, and gross splenomegaly of 20 cm.

Despite broad‐spectrum antibiotic therapy, the patient remained febrile. Blood cultures were unrevealing for any microbial growth. Aphaeretic platelet transfusions were required, despite which he had persistent thrombocytopenia. Prothrombin time/international normalized ratio (PT/INR) was within normal limits, making disseminated intravascular coagulation (DIC) unlikely.

While serum vitamin B12 and serum folic acid levels were normal, hematinic studies were significant for hyperferritinemia with ferritin >16 000 ng/mL, immediately raising the suspicion of HLH. Additional target investigations gave serum triglycerides 5.28 mmol/L (normal 0.3‐1.7), LDH 1807 U/L, and a steep rise of ferritin 75 330 ng/mL within 2 days of diagnosis.

According to the HLH‐2004 trial, this patient fulfilled the diagnostic criteria for HLH, with six out of the eight points: fever *T* > 38.5°C, bicytopenia, splenomegaly on abdominal CT scan, hypertriglyceridemia, and hyperferritinemia. On the *Modified 2009 HLH diagnostic score*, four of four clinical criteria (fever, splenomegaly, cytopenias, and hepatitis) and one of four laboratory criteria (hyperferritinemia) were met. His H score was 258 points, assigning a 99.6% probability of having HLH.

Despite high doses of dexamethasone and etoposide (100 mg/m²) as per the HLH ‐94 treatment protocol, his condition deteriorated further, with worsening respiratory distress, acute liver failure, and progressive confusion. He passed on despite prolonged resuscitative efforts on the 12th day of admission.

By the time the results of the bone marrow trephine biopsy were available, the patient had passed on. The trephine biopsy revealed a diagnosis of EBV‐positive Classic Hodgkin's lymphoma, in addition to CD68 positive scattered histiocytes throughout the bone marrow. In tandem with this, results of EBV serology nuclear antigen (EBNA) IgG were received and were positive at 600 (negative < 5).

### 3.2 Case B

A 47‐year‐old woman presented with a 2‐week history of generalized body weakness. She had been diagnosed with and treated for malaria at a peripheral facility. She also had new onset acute kidney injury, which had been attributed to malaria. Initial assessment at our facility revealed temperature of 38.5°C, HR 100/min, RR 20/min, and BP 155/89 mm Hg. Her abdominal examination was significant for splenomegaly. Initial investigations at presentation are shown in Table 2.

**TABLE 2:** Initial Lab investigations for Patient B

Initial investigation	Value	Reference range
Hemoglobin	7.8 g/dL	11.5‐16.5
MCV	79.4 fL	77‐93
MCH	29.2 pg	27‐33
Hematocrit	13.7%	37‐47
Reticulocyte count	4.49%	0.2‐2
Total leucocyte count	9.7 × 10^9^/L	4‐10
Platelet count	326.00 × 10^9^/L	150‐450
Peripheral blood film	Mild auto agglutination with target cells, basophilic stippling of RBC and left shift of granulocytes. Adequate platelets.	
Lactate dehydrogenase	994 U/L	135‐214
AST	43.6 U/L	0‐35
ALT	16.3 U/L	0‐35
ALP	182 U/L	0‐98
GGT	164 U/L	0‐35
Total bilirubin	28.9 U/L	0‐21
Direct bilirubin	12.9 U/L	0‐3.4
Albumin	26.1 g/L	35‐55
Malaria antigen	Negative	
Malaria parasite	Absent	
Direct Coomb's test	Negative	
Ferritin	1794 ng/mL	13‐150
Folate	10.75 ng/mL	Deficient < 3
Vitamin B12	624.7 pg/mL	191‐663
Serum iron	8.52 Umol/L	6.6‐26
Serum potassium	5.02 mmol/L	3.3‐5.4
Serum creatinine	141 Umol/L	58‐96
Serum bicarbonate	19.1 mmol/L	22‐29
Serum sodium	128 mmol/L	135‐145
Blood urea nitrogen	7 mmol/L	2.1‐7.1
Hepatitis B surface antigen	Positive	
Hepatitis C antibody	Negative	
Hepatitis B viral load	817 IU/mL	
Hepatitis B E‐antigen	Negative	

During the initial workup for anemia, ferritin was noted to be markedly elevated at 1794 ng/mL, which prompted further workup for possible inflammatory causes, namely bacterial infection, connective tissue disease and though lower on the list of possibilities, HLH.

Investigations for an infectious etiology revealed a negative malaria screen, elevated C‐reactive protein (CRP) level 82 mg/L, rising serum lactate dehydrogenase (LDH) to 2149 U/L, and blood cultures unrevealing of any microbial growth.

The connective tissue screen revealed a positive antinuclear antibody (ANA) in a speckled pattern: titer 1:160, reduced levels of complement C3, while C4 was low normal at 0.11 g/L (normal 0.1‐0.4). Anti‐ds‐DNA and ENA were negative. Urinalysis revealed proteinuria of 3+. This gave her a score of 13 on the 2018 SLE criteria from EULAR (European League Against Rheumatism) and ACR (American College of Rheumatology). The criteria require that a patient have ANA titer of at least 1:80 along with 10 points from the clinical and laboratory criteria for a diagnosis of SLE.

A few days after admission, the ferritin level had risen from 1794 to 6929  ng/mL on admission and there was hypertriglyceridemia of 3.24 mmol/L (normal 0.3‐1.7) and falling platelets to a nadir of 103.00 × 10^9^/L.

A CT scan of the chest and abdomen revealed mild hepatomegaly and splenomegaly of 15 cm with subcentimeter nodes above and below the diaphragm.

A bone marrow examination performed on the fifth day of admission revealed hypercellular marrow with features of erythroid hyperplasia and reactive histiocytosis.

According to the HLH‐2004 trial, this patient fulfilled the criteria for a diagnosis of HLH, with five features out of the eight‐point diagnostic criteria: persistent fever > 38.5°C, bicytopenia, splenomegaly on an abdominal CT scan, hypertriglyceridemia, and hyperferritinemia. *Modified 2009 HLH diagnostic criteria*: four of four clinical criteria (fever, splenomegaly, cytopenias, and hepatitis), and one of four laboratory criteria (hyperferritinemia) were met. Her H score was *193* assigning a probability of 82.9% of having HLH.

A diagnosis of HLH due to SLE was made.

Treatment with high doses of steroids was initiated (methylprednisone 500 mg once a day for 3 days), and while she was clinically stable, laboratory parameters worsened, with rising ferritin from 4433 to 6929 ng/mL and falling platelets and LDH levels from 1520 to 2149 U/L. In view of this, a weekly course of rituximab, which is recommended in complicated SLE, was initiated, along with tenofovir, for the chronic hepatitis B infection.

She responded well to this therapy, with resolution of the fever and reduction of the inflammatory markers. Subsequently, she was started on definitive treatment for SLE with hydroxychloroquine and mycophenolate mofetil, and continues to do well on this therapy 10 months later.

### 3.3 Case C

A 25‐year‐old male presented with a 4‐week history of weight loss, fevers, fatigue, diarrhea, vomiting, and abdominal pain. He had been treated at a peripheral facility with artemether lumefantrine for suspected malaria, and also received broad‐spectrum antibiotics (ceftriaxone and doxycycline).

In our emergency room, he was found to be pale and had scleral icterus. Initial assessment revealed temperature 38.7°C, HR 87/min, RR 20/min, and BP 90/58 mm Hg. Apart from hepatosplenomegaly, the rest of the physical examination was unremarkable. Initial investigations at the time of the presentation are shown in Table 3.

**TABLE 3:** Initial Lab investigations for Patient C

Initial investigation	Value	Reference range
Hemoglobin	10.8 g/dL	11.5‐16.5
MCV	78 fL	77‐93
MCH	27pg	27‐33
Hematocrit	31.4%	37‐47
Reticulocyte count	2.37%	0.2‐2
Total leucocyte count	1.49 × 10^9^/L	4‐10
Platelet count	5 .00 × 10^9^/L	
Peripheral blood film	Mild anisopoikilocytosis, marked leukopenia and markedly reduced platelets. No schistocytes	
Lactate dehydrogenase	1140 U/L	135‐214
Fibrinogen	1.1 g/L	2‐4
AST	299.4 U/L	0‐35
ALT	38 U/L	0‐35
ALP	346 U/L	0‐98
GGT	213 U/L	0‐35
Total bilirubin	20.6 U/L	0‐21
Direct bilirubin	15.5 U/L	0‐3.4
Albumin	22.2G/L	35‐55
Malaria antigen	Absent	
Malaria parasite	Absent	
Ferritin	66567 ng/mL	13‐150
Serum iron	14.8 Umol/L	6.6‐26
Serum potassium	4.61 mmol/L	3.3‐5.4
Serum creatinine	79 Umol/L	58‐96
Serum bicarbonate	22.6 mmol/L	22‐29
Serum sodium	131 mmol/L	135‐145
Blood urea nitrogen	9.5 mmol/L	2.1‐7.1
Hepatitis B surface antigen	Negative	
Hepatitis C antibody	Negative	
HIV	Negative	

**FIGURE 1:**
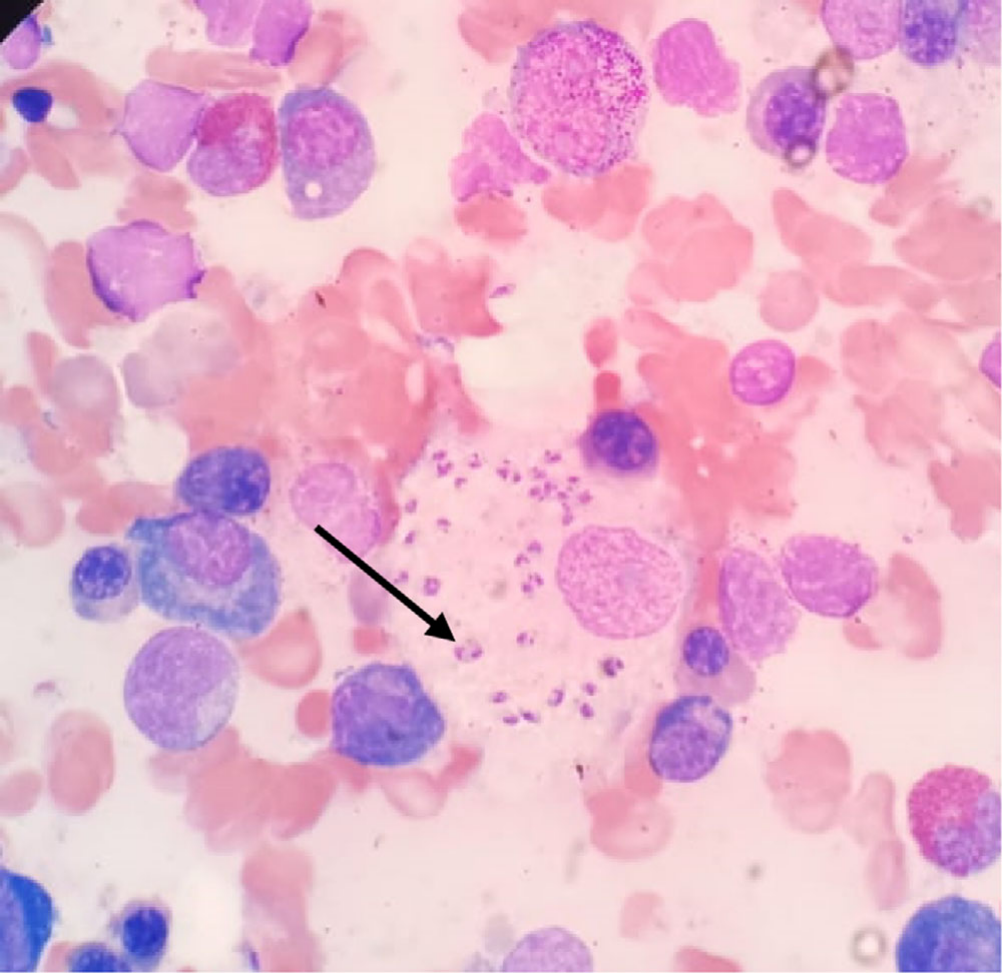
*Leishmania donovani* bodies in bone marrow aspirate

**FIGURE 2:**
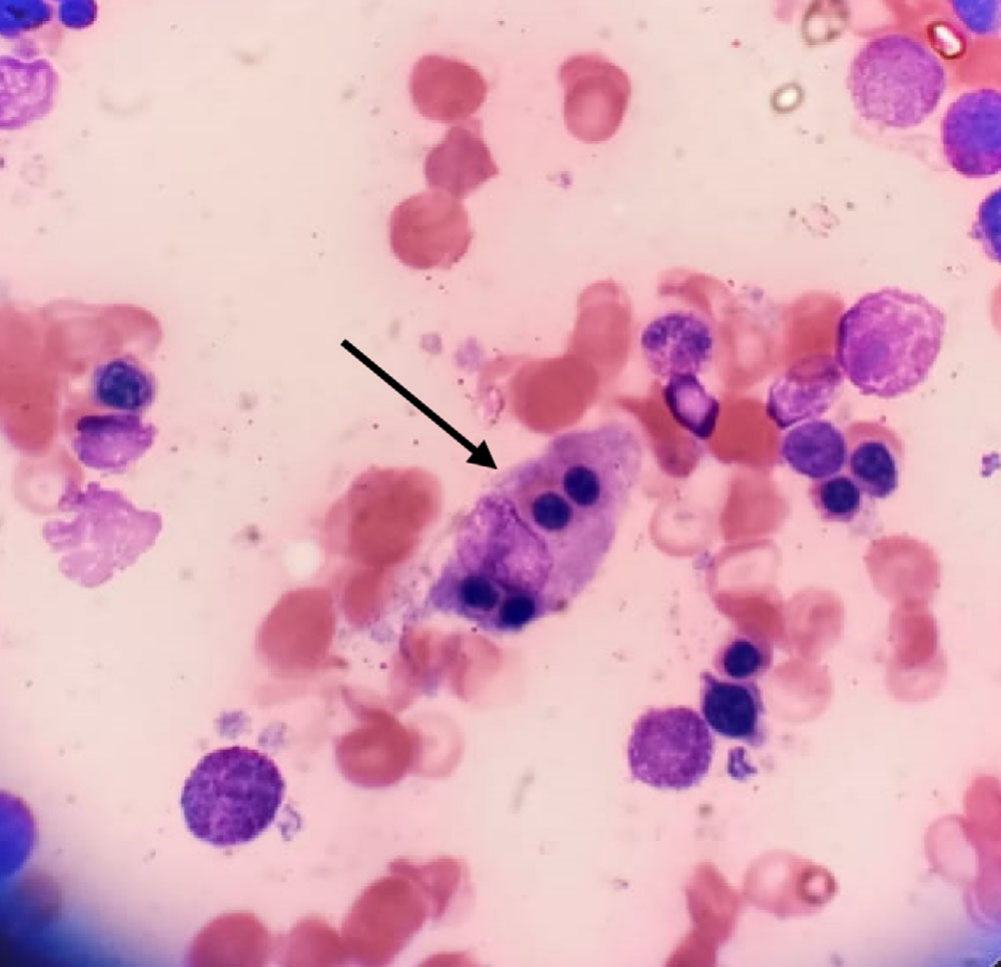
Hemophagocytosis of erythrocytes inside the macrophage

He received fluid resuscitation and was admitted to the high dependency unit (HDU) with a working diagnosis of septic shock and treatment commenced with broad‐spectrum antibiotics. A bone marrow biopsy performed on the first day of admission for severe pancytopenia revealed extracellular and intracellular *Leishmania donovani* bodies, increased histiocytic activity, and evidence of hemophagocytosis (Figures 1 and 2).

His H score was 303 points assigning a 99.97% probability of having HLH. According to the HLH‐2004 trial, this patient fulfilled the diagnosis, with six features out of the eight‐point diagnostic criteria for HLH: persistent fever > 38.5°C, pancytopenia, splenomegaly, hypertriglyceridemia of 5.22 mmol/L, hyperferritinemia, and increased histiocytic activity on a bone marrow aspirate, on which there was also evidence of *L. donovani* bodies, clinching the overall diagnosis; HLH was secondary to visceral leishmaniasis.

He was started on liposomal amphotericin B for 10 days alongside pulsed methylprednisone at 250 mg daily for 4 days, despite which he developed worsening respiratory distress that progressed to ARDS, requiring intubation and mechanical ventilation. He developed DIC, and blood stained endotracheal aspirates, which coupled with increasing ventilator requirements, were suggestive of alveolar hemorrhage.

His stay in the ICU was complicated by multiorgan dysfunction secondary to *Klebsiella pneumonia*e and coagulase negative staphylococcal septicemia, acute kidney injury, and refractory DIC, which failed to respond to aggressive treatment, leading to his demise 15 days after admission.

### 3.4 Case D

A 27‐year‐old patient presented with fever and chills, headaches, shortness of breath on exertion, nausea, and joint pains of 2 days duration. She had been treated for presumed malaria a week prior to her presentation to us.

Clinical examination at admission was significant for temperature 38°C, dark macular patches over the abdominal wall and limbs, the latter of which had been present for about 10 months, and hepato‐splenomegaly. Initial laboratory investigations at the time of the presentation are shown in Table 4.

**TABLE 4:** Initial laboratory investigations for patient D

Initial investigation	Value	Reference range
Hemoglobin	4.2 g/dL	11.5‐16.5
MCV	75 fL	77‐93
MCH	25 pg	27‐33
Hematocrit	13.7%	37‐47
Reticulocyte count	11.54%	0.2‐2
Total leucocyte count	18.58 × 10^9^/L (neutrophilia, bicytopenia, and no schistocytes)	4‐10
Platelet count	65.00 × 10^9^/L	
Peripheral blood film	Mild autoagglutination, anisopoikilocytosis, spherocytes, and reduced platelets.	
Lactate dehydrogenase	1669 U/L	135‐214
Fibrinogen	3.04 g/L	2‐4
AST	146.60 U/L	0‐35
ALT	19.1 U/L	0‐35
ALP	54 U/L	0‐98
GGT	30 U/L	0‐35
Total bilirubin	14.1 U/L	0‐21
Direct bilirubin	5.3 U/L	0‐3.4
Albumin	33.7 g/L	35‐55
Malaria antigen	Positive	
Malaria parasite	Absent	
Direct Coomb's test	Positive	
Ferritin	54 396 ng/mL	13‐150
Folate	>20 ng /mL	Deficient < 3
Vitamin B12	376 pg/mL	191‐663
Serum iron	14.8 Umol/L	6.6‐26
Serum potassium	4.47 mmol/L	3.3‐5.4
Serum creatinine	86 Umol/L	58‐96
Serum bicarbonate	15 mmol/L	22‐29
Serum sodium	128 mmol/L	135‐145
Blood urea nitrogen	7 mmol/L	2.1‐7.1

She gave a history of having sought medical attention for joint pains a year previously, but had not received specific treatment for this suspected arthritis. At the time, she had mild anemia (hemoglobin 10 g/dL), for which iron supplementation had been recommended.

Investigation of the severe anemia revealed a positive direct Coomb's test and an elevated LDH 1660 IU, initially suggestive of hemolysis. However, the hematinic screen gave marked hyperferritinemia, of >50000 ng/mL, which was alarming and prompted a diagnostic workup for HLH. This gave serum fibrinogen level 3.04 g/L, and serum triglyceride level of 3.88 mmol/L, while serum LDH rose to 3965 U/L.

Due to fever, elevated CRP 72 mg/mL, and procalcitonin 10.5 ng/mL, broad‐spectrum antibiotics were initiated. The quantitative buffy coat test for malaria was positive and so she was initially managed for malaria, with intravenous artesunate. However, both thick and thin films were subsequently negative for malaria parasite within 24 h of admission, as was the malaria antigen test. This, and the fact that there was no history of travel to a malaria endemic area, made the diagnosis of malaria unlikely.

Blood cultures assessed after 5 days were unrevealing for any microbial growth. Bone marrow biopsy was performed on the fourth day of admission, which showed increased hemophagocytic activity corroborated by an increase in CD68 positive histiocytes. Given the longstanding joint pains and skin lesions, an autoimmune screen was requested.

According to the HLH‐2004 trial, this patient fulfilled the diagnosis, with six features out of the eight‐point diagnostic criteria for HLH, including a persistent fever >38°C, bicytopenia, splenomegaly, hypertriglyceridemia, hyperferritinemia, and a histiocytic activity on a bone marrow aspirate. Her H score was 243 points assigning a 99.1% probability of having HLH.

She was clinically stable, reporting that she felt better within 48 h of admission. However, transient confusion was noted, overnight, which had settled by the following morning.

Four days into her admission, and after the first dose of methylprednisolone and red cell transfusion, she suffered a tonic clonic seizure, suffered a cardiac arrest, and died despite prolonged resuscitation attempt.

Results of the autoimmune screen were obtained 1 day later, and gave positive ANA in a speckled pattern, negative double‐stranded DNA (dsDNA), while the extractable nuclear antigen was positive for both anti‐Sm and anti‐RNP, clinching the diagnosis in this case to be HLH in a patient with untreated SLE.

Transient confusion while on the ward coupled with a seizure prior to death are highly suggestive of CNS HLH, which carries a dire prognosis.

## 4 SUMMARY OF RESULTS

The age of the patients ranged from 25 to 47 years (male to female ratio 1:1). Fever of undetermined origin, cytopenias, hyperferritinemia, and either splenomegaly or hepatosplenomegaly were prominent features at presentation in each of the cases. All patients met the modified HLH diagnostic criteria according to the HLH‐2004 guidelines that require five out of eight criteria to qualify for a diagnosis of HLH. The clinical and laboratory characteristics are summarized in Table 5.

**TABLE 5:** This table provides the demographic and clinical characteristics of the four patients at the time of diagnosis with HLH

CASE	A	B	C	D
Gender	Male	Female	Male	Female
Time of onset of symptoms to referral	2 months	2 weeks	4 weeks	1 week
Age at diagnosis	30	47	25	27
Fevers	Yes	Yes	Yes	Yes
Splenomegaly	Yes	Yes	Yes	Yes
Cytopenias	Pancytopenia	Bicytopenia	Pancytopenia	Pancytopenia
Ferritin > 500 ng/mL	16 731	1794	66 567	54 396
Triglyceridemia > 3 mmol/L	5.28	3.24	3.78	3.88
Transaminitis	Yes	yes	Yes	Yes
Hemophagocytosis on bone marrow	Yes	No	Yes with *Leishmania donovani* bodies	Yes
HLH 2004 sore	6	5	6	6
H score	258	193	303	243
Associated Infections/malignancy/autoimmune condition	Classic Hodgkin's lymphoma	SLE	Leishmaniasis	SLE
Type of therapy	Etoposide and dexamethasone	Methylprednisone and rituximab	Amphotericin B and methylprednisone	Methylprednisone
Outcome	Dead	Alive	Dead	Dead

Abbreviation: HLH, hemophagocytic lymphohistiocytosis; SLE, systemic lupus erythematosus.

All patients had deranged liver transaminases at presentation with elevated AST levels. Three patients had hemophagocytosis on bone marrow, while the fourth had an increase in the number of histiocytes. *Leishmania donovani* bodies were present in the bone marrow of one patient alongside the hemophagocytosis. Soluble IL 2 and NK cell testing are not available in our country hence were not tested.

A secondary cause of the HLH was identified in all of the patients; two had previously undiagnosed and untreated SLE, one visceral leishmaniasis and the fourth classic EBV‐driven Hodgkin's lymphoma.

## 5 DISCUSSION

HLH is a syndrome of excessive inflammation and tissue destruction due to abnormal immune activation. HLH is on the spectrum of macrophage activating syndromes, in which over activation of macrophages leads not only to exaggerated cytokine production, but also to hyperferritinemia, in the release of iron from macrophages, and hemophagocytosis of blood cells [5, 6, 7, 8].

The sustained activation of macrophages, NK cells, and cytotoxic T cells leads to excessive cytokine production. The persistent production of cytokines, including interferon gamma, tumor necrosis factor alpha, interleukins (ILs) such as IL‐6, IL‐10, and IL‐12, and the soluble IL‐2 receptor, further recruits additional inflammatory cells, in a vicious cycle, leading to a cytokine storm. This cytokine storm is the primary mediator of tissue damage [9].

Macrophages are a storage reservoir of ferritin, and this accounts for high ferritin levels in HLH [10]. Recorded peak ferritin levels greater than 10 000 g/L was found to have a 90% sensitivity and 96% specificity for HLH during a study done at the Texas Children's Hospital [11]. Therefore, when the clinical and laboratory features are suggestive, a serum ferritin of >10 000 g/L strongly supports a diagnosis of HLH [2]. This was certainly reflected in three of four cases of this series, where ferritin, initially performed in assessment of anemia, unexpectedly gave hyperferritinemia > 10 000 g/L, immediately triggering investigations for HLH.

The most common presentation of HLH as per the HLH‐2004 study included fever, splenomegaly, bicytopenia, hypertriglyceridemia or hypofibrinogenemia, hemophagocytosis, ferritin > 500 mcg/L, low or absent NK cell activity, and soluble CD25 elevation [12]. As summarized above, all four patients described in this series presented with fever, splenomegaly, hyperferritinemia, and cytopenias.

In the early stages of HLH, hemophagocytosis might not be seen in the bone marrow. This and the patchy nature of this phenomenon in bone marrow samples mean that its absence does not negate HLH as the diagnosis. The incidence of bone marrow involvement varies between 25% and 100% [13] and bone marrow analysis for presence of hemophagocytosis has a sensitivity of around 60% [14]. One of the patients (Case B) did not have hemophagocytosis on the bone marrow, but did have an excess of histiocytes within the bone marrow. This probably corresponds with the patient having the "mildest" and possibly earliest form of HLH in the cohort and hence being picked up at the earlier than the other cases.

The overall mortality of HLH is high, varying between 26.5 and 74.8%, and depends on the patient characteristics, underlying precipitating conditions, and stage of disease [15]. This was also seen in our case series, which reported a high mortality rate, with the only survivor having been diagnosed at a relatively early stage of the disease course, and remaining in good health to date.

### 5.1 Diagnostic criteria

The diagnostic criteria is based on the HLH‐2004 trial [12, 16]. To make a diagnosis, at least five out of eight findings are required as highlighted in Tables 6 and 7. All the patients in this case series (100%) fulfilled both the 2004 and the modified 2009 HLH criteria. Additionally, the HLH H score was calculated for each patient and gave a high probability of HLH in all the patients (99% for cases A, C, D, and for 82.9% for case B). The H score is a weighted criteria score, validated for diagnosis in secondary adult causes of HLH. It is aimed at achieving a more effective estimate of the probability of HLH and consists of graded clinical and laboratory parameters with a sensitivity and specificity of 90% and 79% [17]. In a retrospective study done to compare the performance of HLH‐2004 guidelines with the new H‐score, H‐score performed better in identifying HLH at presentation among adult patients [15, 18, 19].

**TABLE 6:** HLH diagnostic criteria; at least five of eight findings present

1.	Fever ≥38.5°C
2.	Splenomegaly
3.	Ferritin > 500 ng/mL
4.	Peripheral blood cytopenias, with at least two of the following: hemoglobin < 9 g/dL; platelets < 100.00 × 10^9^/L; absolute neutrophil count < 1 × 10^9^/L
5.	Hypertriglyceridemia (fasting triglycerides > 3 mmol/L) and/or hypofibrinogenemia (fibrinogen < 150 mg/dL)
6.	Hemophagocytosis in bone marrow, spleen, lymph node, or liver
7.	Low or absent natural killer (NK) cell activity
8.	Elevated soluble CD25 (soluble IL‐2 receptor alpha [sIL‐2R]) two standard deviations above age‐adjusted laboratory‐specific norms

**TABLE 7:** Modified 2009 HLH criteria

Clinical	Three of four clinical findings	Fever Splenomegaly Cytopenias Hepatitis
	Plus	
Immune markers	One of four immune markers	Hemophagocytosis Increased ferritin Hypofibrinogenemia Absent or very decreased NK cell function

### 5.2 Triggers of HLH

In all four cases, a trigger for HLH was identified, but in only one of these cases, characterised by early patient presentation, timely clinical suspicion, work up and treatment resulted in a favourable patient outcome. Macrophage activation has been reported to be triggered by infections, most commonly viral infections such as EBV [20, 21, 22], and has also been associated with leishmaniasis and CMV [22]. Malignancies, most commonly lymphoproliferative, are also recognized to be activators of the immune system as are autoimmune conditions. Although a seemingly heterogeneous group of conditions, the disorders mentioned above all have the ability to activate the immune system and lead to a macrophage activating syndrome typical of HLH, characteristic of the disordered, exaggerated response, and its resultant tissue damage.

### 5.3 Treatment

Therapy is based on the HLH‐94 protocol [23, 24]. The standard treatment is corticosteroids, to which etoposide is added in cases which do not initially respond, and in which no other cause is found for the HLH, along with identification and treatment of any underlying cause. Rituximab is recommended in EBV‐driven HLH, with the mechanism of action being eradication of the EBV reservoir through clearance of B cells [15, 19]. For central nervous system disease, intrathecal methotrexate and hydrocortisone are added to the induction therapy [24]. In patients not showing signs of improvement, the above therapy is continued while planning for allogeneic hematopoietic cell transplantation in suitable cases.

Patient A had EBV‐driven classical Hodgkin's lymphoma but the diagnosis was only available postmortem. Additionally, he had been unwell for a number of months prior to presentation. Despite initiating etoposide and methylprednisolone within days of presentation at our center, he had a rapid clinical deterioration and died. Rituximab therapy would have been a recommendation in his case, had EBV PCR been performed and found positive [15, 19]. HLH associated with lymphomas tends to have a higher mortality compared to HLH due to infections and autoimmune conditions [22]. Worsening cytopenias and cholestasis are predictive of death in HLH, and a rapid drop in ferritin levels after initiation of treatment is associated with improved outcome [25].

Both patients B and D had SLE. However, while patient B had a history of symptoms that had been present for approximately 1 month, patient D had symptoms for more than 10 months, and indeed had been evaluated and found to have joint pains, anemia, and skin lesions at that time, but had been lost to follow up. Both patients had ferritin assessment early on as part of the investigation of the cause of anemia at presentation. Patient B's ferritin level was 1794 ng/mL, while patient D's ferretin level was 54 396 ng/mL.

A high index of suspicion of HLH in patient B, mainly because there were no other significant pointers to an alternative pathology led to early initiation of high‐dose methylprednisolone, and subsequent positive ANA serology allowed for definitive treatment of SLE. This patient responded well to treatment and is now asymptomatic, more than a year later.

Although patient D's elevated ferritin was also investigated promptly, hyperferritinemia > 50 000 at presentation is indicative of already established macrophage activation, which could not be reversed even with timely treatment. Additionally, the fact that she had transient neurological symptoms and a generalized seizure is highly suggestive of neuro‐HLH, which carries a grave prognosis.

Patient C had a bone marrow diagnosis of leishmaniasis, coupled with features of advanced HLH. However, he too presented several weeks after falling ill, and receiving antibiotic therapy for presumed bacterial infection at another center. Despite high dose of methylprednisolone and amphotericin B, it was impossible to reverse the established HLH and multiorgan failure, which were present at presentation, leading to his adverse outcome.

## 6 CONCLUSION

Diagnosis of HLH presents a diagnostic challenge to clinicians due to the nonspecific signs and symptoms that portend a wide array of differential diagnosis. Therefore, a high index of suspicion is warranted especially when a patient presents with unremitting fevers and cytopenias. In our series, an unexpectedly high ferritin, which in each case was performed early in course of admission to investigate anemia, resulted in early suspicion of the condition and relatively early appropriate investigations and treatment for HLH.

However, despite benefitting from an early diagnosis and initiation of therapy for HLH, vis‐a‐vis their admission to our facility, three of these patients presented with already established macrophage activation, HLH, and features of organ failure. Therefore, the HLH‐directed treatment, though appropriate, proved futile in these cases.

The mortality of patients with HLH is very high and delay in diagnosis confers the greatest risk to a fatal outcome, as shown in this series where the time to presentation ranged from 2 weeks to 2 months.

This case series highlights the difficulty in obtaining a good outcome in HLH, even when the diagnosis is suspected early on. Additionally, HLH was not suspected at peripheral facilities, probably due to a combination of factors: (a) it is uncommon and shares features with sepsis, which is far more common; (b) ferritin level, which was markedly elevated and prompted a search for HLH at our center, is not widely available at peripheral facilities and can be costly.

It cannot be stressed enough that a high index of suspicion is warranted, when faced with a patient with a triad of fever, cytopenias, and organomegaly, which is unexplained, and that checking a ferritin level in this case may highlight unexplained hyperferritinemia and lead to appropriate investigations for HLH. The patients described here were diagnosed with HLH promptly within our service, partly because we were able to quickly discount alternative diagnoses as treatment of these had already been initiated with poor outcome at peripheral facilities.

A multidisciplinary approach, including early consultations with a haematologist, either remotely or in person is essential to promptly investigate and initiate appropriate therapy, which is relatively accessible. Prompt, lifesaving and timely treatment, is likely to have to be initiated before all test results have been returned, hence relying on a consensus of clinical and laboratory features compatible with the diagnosis, but not **yet** diagnostic of it.
